# Case of Spinal Irritation Giving Rise to Symptoms Simulating Catalepsy

**Published:** 1843-10-28

**Authors:** George L. Upshur

**Affiliations:** Norfolk, (Va.)


### A CASE OF SPINAL IRRITATION GIVING RISE TO SYMPTOMS SIMULATING CATALEPSY.

BY GEO. L. UPSHUR, M. D.

William S——, coach-maker's apprentice, aged 20, of a lymphatic habit, was seized on the night of the first of October, with total insensibility, the want of animation being so complete that his friends thought he was dead, and had made preparations to shroud him. He remained in this state about two hours. I saw him early on the following morning, when his condition was as follows:—skin cool; pulse 90, soft and full; slight cephalalgia; pupils somewhat contracted; tongue clean, pale, and flabby; countenance expressive of languor; bowels constipated; appetite not disturbed; no tympanitis nor thirst; complained of frequent palpitations, accompanied by sharp, darting pains, alternately, on either side of the thorax; action of the heart preternaturally quick,—sounds normal; no alteration in the respiratory murmur. I then commenced an examination of the spinal column, beginning at the occipital ridge and making uniform pressure to the os coccygis. At the seventh cervical, and again at the last dorsal vertebræ, the patient cried out upon the slightest pressure being applied. He was not aware of the existence of these tender places until I made the examination. On the night of the 2d inst. he had another cataleptic paroxysm, being unconscious for the space of an hour. I did not see him during this attack, but was informed by his friends, that throughout the whole of it his eyes were closed, with the balls permanently fixed—his breathing was scarcely perceptible—had no flushing of the face, nor inordinate action of the carotids; he suffered himself to be placed in any position without manifesting the least volition, and could not be aroused, either by loud noises, or the application of irritants to different parts of the surface. Consciousness returned with frequent sighs and an appearance of great debility. Upon being questioned, he was ignorant that any thing wrong had happened, and resumed the subject of conversation with which he had been engaged just prior to the paroxysm.

*Treatment.* After the first attack the patient was ordered ℥ij. ol. ricini, and the tender places in the back to be frequently rubbed with a liniment composed of ol. tiglii, ol. terebinthinæ, tinct. canth., and aq. ammon. After the second paroxysm he took one drop of tiglii with five grains of calomel; and eight cut cups were applied along the dorsal vertebræ, to be followed by a blister 3 in. by 6. In four days the blister healed, and the soreness had entirely disappeared. He is now well and has had no return of the catalepsy, nor other unpleasant sensations, since the application of the cups and blister.

*Remarks.*—I look upon this case as not entirely devoid of interest. The true pathology of catalepsy, as well as that of tetanus, epilepsy, and several other nervous diseases, has never been satisfactorily determined. Autopsies throw little or no light upon the subject, for these diseases, (especially catalepsy,) are so rare that one, in an extensive practice, may pass a whole professional life without witnessing a single case. The causes of catalepsy, as mentioned in the works of authors, are numerous, but I have no where seen spinal irritation mentioned as one of them. The cause of the peculiar phenomena in catalepsy, beyond a doubt, is to be sought for in the cerebro-spinal centres as is demonstrated by the complete lack of energy on the part of the brain, and the condition of the muscular system, during each attack. Spinal irritation first makes its appearance by the venous sinuses about the roots of the nerves becoming congested; this is rapidly followed by irritation of the adjacent neurilemes, May not the state of the brain in catalepsy be legitimately derived from this irritation of the nervous sheaths, upon the principle of reflex nervous action? A slight puncture of the smallest nervous filament in one of the extremities will not unfrequently develope tetanic spasms.

Richter, as quoted by Eberle, has recorded a case of intermitting fever, where every paroxysm was ushered in by cataleptic seizure. In the case above detailed, I was at first disposed to attribute the catalepsy to the same cause, but, upon further inquiry was informed that the patient had been subject to similar attacks for six months, without any tendency to regularity in their occurrence: that he would sometimes have three fits in the course of twenty-four hours, and then perhaps a whole week would elapse without a single one. The non-appearance of fever after the subsidence of each attack, would also disprove the intermittent tendency.

I report this case, not only as something anomalous, but also because it is calculated to throw some light upon the diagnosis and pathology of nervous diseases. Patients often present themselves to us, complaining of darting pains in different parts of the body; palpitation; gastric disturbance; suppression of the urinary, or other visceral secretions; and of a variety of other unpleasant sensations, more or less well marked, all of which may be traced to a single tender place along the spine. Indeed, the symptoms which arise in some cases of spinal irritation are so numerous and so severe, as to distract the attention of even the most cautious physician from the true seat of the disease, and the treatment, in consequence, is directed into the wrong channel.

*Norfolk*, (*Va.*) *Oct.* 23*d*, 1843.

